# Relationship of clusterin with renal inflammation and fibrosis after the recovery phase of ischemia-reperfusion injury

**DOI:** 10.1186/s12882-016-0348-x

**Published:** 2016-09-20

**Authors:** Jia Guo, Qiunong Guan, Xiuheng Liu, Hao Wang, Martin E. Gleave, Christopher Y. C. Nguan, Caigan Du

**Affiliations:** 1 Department of Urologic Sciences, University of British Columbia, Vancouver, BC Canada; 2 Department of Urology, Renmin Hospital of Wuhan University, Wuhan, Hubei China; 3 Department of General Surgery, Tianjin Medical University General Hospital, Tianjin, China; 4 Vancouver Prostate Centre, Vancouver, BC Canada; 5 Department of Urologic Sciences, The University of British Columbia, VGH-Jack Bell Research Centre, 2660 Oak St, Vancouver, BC V6H 3Z6 Canada

**Keywords:** Clusterin, Kidney ischemia-reperfusion, Acute kidney injury, Chronic kidney disease, Fibrosis

## Abstract

**Background:**

Long-term outcomes after acute kidney injury (AKI) include incremental loss of function and progression towards chronic kidney disease (CKD); however, the pathogenesis of AKI to CKD remains largely unknown. Clusterin (CLU) is a chaperone-like protein that reduces ischemia-reperfusion injury (IRI) and enhances tissue repair after IRI in the kidney. This study investigated the role of CLU in the transition of IRI to renal fibrosis.

**Methods:**

IRI was induced in the left kidneys of wild type (WT) C57BL/6J (B6) versus CLU knockout (KO) B6 mice by clamping the renal pedicles for 28 min at the body temperature of 32 °C. Tissue damage was examined by histology, infiltrate phenotypes by flow cytometry analysis, and fibrosis-related gene expression by PCR array.

**Results:**

Reduction of kidney weight was induced by IRI, but was not affected by CLU KO. Both WT and KO kidneys had similar function with minimal cellular infiltration and fibrosis at day 14 of reperfusion. After 30 days, KO kidneys had greater loss in function than WT, indicated by the higher levels of both serum creatinine and BUN in KO mice, and exhibited more cellular infiltration (CD8 cells and macrophages), more tubular damage and more severe tissue fibrosis (glomerulopathy, interstitial fibrosis and vascular fibrosis). PCR array showed the association of CLU deficiency with up-regulation of CCL12, Col3a1, MMP9 and TIMP1 and down-regulation of EGF in these kidneys.

**Conclusion:**

Our data suggest that CLU deficiency worsens renal inflammation and tissue fibrosis after IRI in the kidney, which may be mediated through multiple pathways.

**Electronic supplementary material:**

The online version of this article (doi:10.1186/s12882-016-0348-x) contains supplementary material, which is available to authorized users.

## Background

Ischemic acute kidney injury (AKI) occurs when renal perfusion is decreased [1, 2], and it is the most common form of clinical AKI, accounting for up to 70 % of all AKI cases [3–6]. With adequate therapy, quick recovery of kidney function to baseline may occur and is considered a hallmark of ischemic AKI [6, 7]. Many recent studies have documented long-term sequelae following AKI, including: increased risk of developing chronic kidney disease (CKD) [8–12], worsening of preexisting CKD [8, 13], and progression to end-stage renal disease (ESRD) [8, 12, 14]. Increasing evidence in literature suggests that many pathological factors including microvascular rarefaction, hypoxia, inflammation and renal tubular injury play an important role in the progression of CKD [15–20], but the precise pathophysiologic pathways mediating the progressive insults associated with AKI leading to CKD are still not fully understood, and thus there are currently no viable therapeutic options available to prevent CKD progression after renal injury.

Clusterin (CLU) is a 75–80 kDa disulfide-linked heterodimeric glycoprotein that is encoded by a single gene in both the human and mouse genomes [21, 22]. This protein not only is a major glycoprotein in physiological fluids, such as plasma, milk, urine, cerebrospinal fluid, and semen [21], but is also upregulated in renal tissues of both humans and experimental models by various forms of cellular stress, such as unilateral ureteral obstruction (UUO) [23], ischemia-reperfusion injury (IRI) [24–26], as well as in rejected renal allografts and those native kidneys with intrinsic renal disease [27–29]. However, the biological importance of CLU for the kidney is not fully understood. We and others have been investigating the biological impacts of CLU deficiency on adult kidneys using CLU knockout (KO) mice compared to CLU-expressing wild type (WT) control mice, and have demonstrated: (i) CLU KO in aging mice results in developing progressive glomerulopathy that is associated with glomerular antibody deposition [30]; (ii) CLU KO mice exhibit the higher levels of renal fibrosis in response to ureteral obstruction [31] or angiotensin II stimulation [32]; and (iii) our group has shown that CLU KO results in more severe renal IRI [26], in an experimental model of ischemic AKI, and also impairs renal repair after IRI [33]. However, the role of CLU in the progression to CKD from AKI has not been investigated yet. In the present study, the effects of CLU deficiency on the transition of ischemic AKI to CKD were investigated using CLU KO mice in a model of renal IRI compared to WT controls.

## Methods

### Animals

Both wild type (WT) C57BL/6 (B6) and CLU knockout (KO) mice in B6 background (B6-Clu^−/−^) were received from the breeding colonies in the animal facility at the Jack Bell Research Centre (Vancouver, BC, Canada). All the animals (males, 12- weeks old) for the experiments were cared in accordance with the Canadian Council on Animal Care guideline under the protocols approved by the Animal Use Subcommittee at the University of British Columbia (Vancouver, BC, Canada).

### Renal IRI

Unilateral renal IRI was induced in the left kidney of WT versus CLU KO mice following a routine surgical procedure in the lab. In brief, mice were anesthetized with the combination of ketamine (100 mg/kg) and xylazine (10 mg/kg), and isoflurane as needed. The left kidneys were exposed through a flank incision, followed by the induction of ischemia in these kidneys through clamping renal pedicles at the body temperature of 32 °C for 28 min. After the clamps were released, reperfusion of the kidneys was confirmed visually. The non-ischemic right kidneys in the same mice were kept as contralateral controls as well as for life support.

### Determination of renal function

The function of the left kidneys after 14 or 30 days of IRI was determined by using serum levels of both creatinine and blood urea nitrogen (BUN). In brief, the contralateral/right kidney was removed on day 13 or 29 after IRI, after 24 h of right kidney removal, mice were euthanized and serum samples were collected. Both creatinine and BUN in the serum samples were measured in the Chemistry Laboratory at the Vancouver Coastal Health Regional Laboratory Medicine (Vancouver, BC, Canada) by using the Dimension Vista® System with CRE2 and BUN Flex® reagent cartridges (Siemens Healthcare Diagnostics Inc., Newark, DE, USA), respectively.

### Isolation of infiltrating leukocytes and splenocytes

The procedure of infiltrate isolation from the kidneys or splenocytes from the spleens was described in our previous studies [34, 35]. Mice were randomly selected from each group for this experiment. In brief, after perfusion with phosphate buffered saline (PBS), kidney tissues were minced and digested with collagenases D (Worthington Biochemicals, Lakewood, NJ, USA). The resultant cell suspensions were filtered through 30-μm nylon mesh, and leukocytes from the cell suspension were recovered from interphase by a density centrifugation using 1.083 g/ml Histopaque (Sigma-aldrich Canada, Oakville, ON, Canada), and were finally suspended in PBS after washing with PBS. A single cell suspension of splenocytes was prepared by gently crushing the spleens of mice in PBS in a Cell strainer (BD — Canada, Mississauga, ON, Canada), followed by removal of erythrocytes by a brief incubation (~4 min) with lysis buffer (0.15 M NH_4_Cl, 1.0 mM KHCO_3_, 0.1 mM EDTA, pH 6.8). After washing with PBS, the splenocytes were finally suspended for examination. The total number of isolated cells from each kidney or spleen was counted using a TC10™ automated cell counter (Bio-Rad Laboratories Canada, Mississauga, ON, Canada) with trypan blue stain.

### Flow cytometric analysis

The phenotypes (CD4^+^, CD8^+^, CD45R/B220^+^, pan NK^+^, CD11b^+^, and Mac3^+^ cells) of leukocyte population in cell suspensions (kidney infiltrates and splenocytes) was determined by fluorescence-activated cell sorting (FACS) analysis using fluorescent-labeled rat monoclonal antibodies [anti-CD4 (clone YTS191-1), anti-CD8A (clone 53-6-7), anti-CD45R/B220 (clone RA3-6B2), anti-pan NK (clone DX5), anti-CD11b (clone M1-70-15), and anti-Mac3 (clone M3-84) antibodies] following manufacture’s protocol (*e*Bioscience, San Diego, CA, USA). Briefly, cells were probed with each fluorescence dye-conjugated antibody in the dark for 30 min at 4 °C. After washing with PBS, the positive stain was counted by using a flow cytometry and further quantified using FlowJo software (Tree Star Inc., Ashland, OR, USA).

### Determination of renal pathologies by histology

Kidneys after IRI insult were randomly selected from each group for histological analyses. A coronal tissue slice through the mid-portion of each kidney was fixed in 10 % neutral buffered formalin, followed by embedded in paraffin wax. Sections were cut at 4-μm thickness and stained with hematoxylin and eosin (HE) for the examination of cellular infiltration, periodic acid-Schiff (PAS) for tubular injury or Masson’s trichrome (MT) method for collagen fiber deposition. The sections were scanned with Leica SCN400 Slide scanner (Leica Microsystens Inc., Concord, ON, Canada). The pathological parameters of renal fibrosis after IRI were determined in two separate sections of each kidney using the Digital Image Hub – A slidepath Software Solution (Leica Microsystems Inc.) in a blinded fashion as described previously [36].

The extent of mononuclear cell infiltration in renal cortex of a kidney was assessed in HE-stained sections using a 0 to 4 scale, depending on the percentage of cellular infiltrates-occupied area in each microscopic view: 0 (normal or no sign of infiltration), 1 (1–10 % of the area affected with cellular infiltration), 2 (11–25 %), 3 (26–50 %), and 4 (>50 %). The average of at least 20 randomly selected views represented the infiltration in each kidney.

The number of injured tubules, including cellular loss (atrophy), intratubular cast formation, tubular cell flattening or vacuolation, in each microscopic view in renal cortex of a kidney was counted in PAS-stained section, and the average number of at least 20 randomly selected views represented the injured tubules in each kidney. The percentage of damaged tubule (combined both necrosis and vacuolization) in total of tubules was counted in each view, randomly selected in the region of renal cortex under 400× magnification (high-powered field – hpf), and was averaged at least of 20 nonoverlapping fields for each kidney.

The severity of renal fibrosis was semi-quantitatively determined by the degree of tubulointerstitial fibrosis in renal cortex of the kidney, the percentage of affected glomeruli (glomerulopathy) and of interlobular arterioles or arteries in Masson’s trichrome-stained sections. The tubulointerstitial fibrosis was determined using a 0 to 4 scale based on the severity of tubular dilation or the percentage of the area stained positively with collagen fibers: 0 (normal interstitium with the absence of collagen fiber stain), 1 (minimal collagen fiber stain or less than 5 % of affected area), 2 (mild or 6–25 %), 3 (moderate or 26–50 %) and 4 (severe, or affecting >50 % of the cortical area). The percentage of affected glomeruli (glomerulopathy), including glomerulosclerosis (focal and segmental sclerosis) and glomerular hypertrophy, was determined in a range of 180 to 250 glomeruli of each kidney. And the percentage of affected interlobular arterioles or arteries, determined by the presence of fibrous intima, was counted in a range of 10 to 20 interlobular arteries or arterioles of each kidney.

### Immunohistochemical analysis

The kidneys were harvested from mice after perfusion with PBS, followed by formalin fixation, paraffin embedding and section as described above. The expression of alpha-smooth muscle actin (α-SMA) protein in kidney sections was assessed by a standard immunohistochemical method. Briefly, after deparaffin and rehydration buffered-formalin-fixed sections were treated with 3 % H_2_O_2_ in Tris buffer saline (TBS) (pH 7.4) for 30 min at room temperature to quench endogenous peroxidase, followed by permeabilization with 0.2 % Triton X-100 for 10 min. After being washed with TBS containing 0.1 % Tween 20 (TBS-T) and blocked with 5 % normal rabbit serum, the sections were incubated with 1:50 dilution of primary mouse monoclonal anti-α-SMA (clone 1A4, Sigma-Aldrich Canada, Oakville, ON, Canada) overnight at 4 °C. The immune complexes of α-SMA and the antibody on the tissue section were detected using Vector M.O.M. Immunodetection kit following manufacturer’s protocol (Vector Labs, Burlington, ON, Canada), and were visualized using a 3,3′-diaminobenzidine (DAB) peroxidase substrate. The control negative staining included the sections incubated with normal mouse IgG instead of mouse anti-α-SMA antibody as the primary antibody. Nuclei are counterstained with hematoxlin.

### Fibrosis PCR array

The expression of 84 fibrosis-associated genes was quantitatively examined in WT kidney tissues compared to CLU KO controls using Mouse Fibrosis RT^2^ Profiler™ PCR Arrays kits following manufacturer’s instruction (SABiosciences – QIAGEN Inc., Valencia, CA, USA). In brief, following perfusion with PBS, one part of kidney tissue was snap-frozen in liquid nitrogen and stored at −80 °C prior to RNA extraction. Four kidney samples from each group were randomly selected for the determination of fibrosis-related gene expression profile. The total RNA from kidney tissues was extracted and purified using the RNeasy Microarray Tissue Mini kit (QIAGEN Canada, Toronto, ON, Canada), and converted to cDNA using RT^2^ First Strand Kit (QIAGEN Canada). The expression of selected genes was amplified by using real-time Mouse Fibrosis RT^2^ Profiler™ PCR Arrays. Data were analyzed using Web-based PCR Array Data Analysis Software (www.SABiosciences.com/pcrarraydataanalysis.php.).

### Statistical analysis

Data were collected from individual experiment or mouse in each study, and were presented as mean ± standard derivation (SD) for each group in the text. The difference between groups was analyzed using *t*-tests or analysis of variance (ANOVA) as appropriate with Prism® software (GraphPad Software, Inc., La Jolla, CA, USA). A *p* value of ≤0.05 was considered significant.

## Results

### Significant reduction of kidney weight is induced by IRI, but not affected by CLU deficiency

**Fig. 1:**
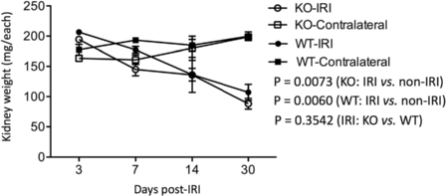
No difference in the progression of kidney atrophy between CLU KO mice and WT controls after IRI. Renal IRI in left kidneys of CLU KO versus WT mice was induced by clamping renal pedicles for 28 min at the body temperature of 32 °C, and the weight of the left kidney and contralateral right kidney from each mouse was recorded at different time points after reperfusion. Data are presented in the figure as mean ± standard error of the mean (SEM) of each group. KO-IRI: the left kidneys of CLU KO mice (*n* = 4–15) after IRI (*p* = 0.0073, one-way ANOVA); KO-Contralateral: the right/contralateral kidneys of CLU KO mice (*p* = 0.0553, one-way ANOVA); WT-IRI: the left kidneys of WT mice (*n* = 4–22) after IRI (*p* = 0.0060, one-way ANOVA); and WT-Contralateral: the right/contralateral kidneys of CLU KO mice (*p* = 0.5140, one-way ANOVA). Kidney atrophy in KO versus WT mice, *p* = 0.3542 (two-way ANOVA)

It has been demonstrated that IRI initiates progressive renal atrophy, indicated by the reduction in renal weight, volume and cortical thickness accompanying tubular cell death (both apoptosis and necrosis) and interstitial infiltration [37–39]. CLU plays an anti-apoptotic or prosurvival role in the kidney against IRI [26, 40]. Surprisingly, the present study showed that the renal atrophy indicated by the loss of renal mass was not statistically different between WT and KO groups (Fig. 1). The kidney weight in WT mice at age of 12 weeks old was 192 ± 20.31 mg, and was not different from 179 ± 17.91 mg in age-matched KO mice (*P* = 0.3741, two-tailed *t*-test, *n* = 4). As shown in Fig. 1, after IRI, the left kidneys in CLU KO mice were 194.5 ± 0.71 mg on day 3 (*n* = 4), 145.0 ± 21.21 mg on day 7 (*n* = 4), 136.0 ± 65.04 mg on day 14 (*n* = 5) and 88.13 ± 34.46 mg on day 30 (*n* = 15) (*P* = 0.0073, one-way ANOVA), while the weight of their contralateral controls was not significantly changed, and it was 163.5 ± 0.71 mg on day 3 (*n* = 4), 161.0 ± 15.56 mg on day 7 (*n* = 4), 180.0 ± 44.72 mg on day 14 (*n* = 5) and 198.67 ± 12.46 mg on day 30 (*n* = 15) (*P* = 0.0553, one-way ANOVA). Similar results were seen in WT mice, in which IRI induced kidney weight loss from 206.5 ± 9.19 mg on day 3 (*n* = 4) to 107.15 ± 62.11 mg on day 30 (*n* = 22) (*P* = 0.0060, one-way ANOVA) (Fig. 1). More importantly, statistical analysis revealed that the atrophic degree or the weight loss of the left kidneys after IRI between CLU KO and WT mice was not significantly different (*P* = 0.3542, two-way ANOVA), demonstrating the occurrence of severe atrophy in the kidney after IRI, but at the same time not significantly affected by a deficiency in CLU expression.

### In CLU KO mice, kidney recovery from IRI occurs after 14 days

**Fig. 2:**
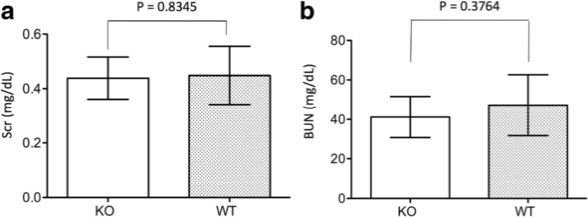
No difference in renal function between CLU KO and WT mice on day 14 after IRI. On day 13 after IRI, contralateral kidneys were removed. After 24 h of surgery, serum from each mouse was collected, and the levels of both serum creatinine (Scr) and blood urea nitrogen (BUN) were measured as biomarkers of renal function. Data are presented as mean ± standard derivation (SD) of each group. **a** Scr in CLU KO group (*n* = 8) compared to WT control (*n* = 8), *p* = 0.8345 (two-tailed *t*-test). **b** BUN in CLU KO group (*n* = 8) compared to WT control (*n* = 8), *P* = 0.3764 (two-tailed *t*-test)

**Fig. 3:**
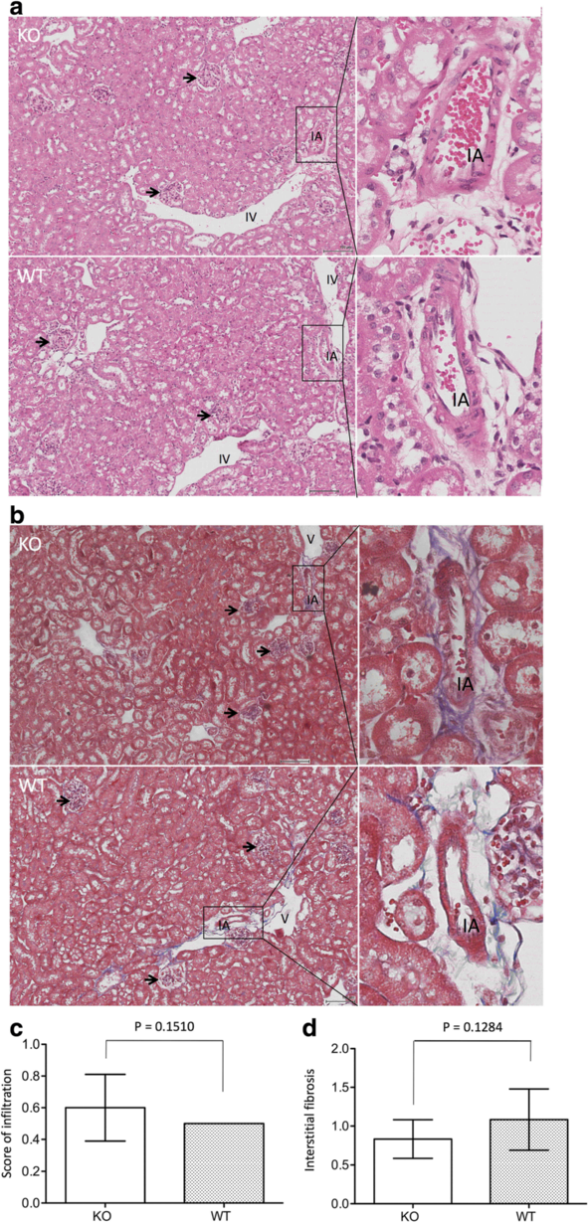
No difference in tissue architecture of the kidneys between CLU KO and WT mice on day 14 after IRI. The sections of the left kidneys of WT versus CLU KO mice, harvested on day 14 after reperfusion, were stained with hematoxylin and eosin (HE) or Masson’s trichrome (MT). Data are presented as a typical microscopic image of renal cortex. KO: CLU null kidney sections; WT: WT kidney sections. **a** Typical microscopic images of HE-stained sections showing intact tubules with minimal cellular infiltration in the perivascular space. **b** Typical microscopic images of MT-stained sections showing red cytoplasm and mild blue collagen in the perivascular space. *Black arrows*: glomeruli; IA: interlobular artery; and IV: interlobular vein. **c** The infiltration was semi-quantitatively scored in two separate sections of each injured left kidney, and was presented in average per view. Data are presented as mean ± SD of each group. KO group vs. WT control: *P* = 0.1510 (two-tailed *t*-test, *n* = 9). **d** The interstitial fibrosis was semi-quantitatively scored in two separate sections of each injured left kidney, and was presented in average per view. Data are presented as mean ± SD of each group. KO group vs. WT control: *P* = 0.1284 (two-tailed *t*-test, *n* = 9)

Our previous study demonstrated there were no signs of tissue repair in the kidneys of CLU KO mice until day 7 after IRI, whereas WT controls showed significant improvement [33]. After induction of IRI in the left kidneys under the same conditions as performed in the previous study, kidney atrophy was no different between WT and KO group on day 14 [CLU KO: 136.0 ± 65.04 mg (*n* = 5) vs WT: 138.36 ± 34.73 mg (*n* = 11) (*P* = 0.9885, two-tailed *t*-test)]. Similarly, the function of these kidneys was not different, indicated by the fact that after removal of contralateral kidneys the serum levels of creatinine or BUN in KO mice were 0.44 ± 0.08 mg/dL (*n* = 8) or 41.17 ± 10.42 mg/dL (*n* = 8), which were not statistically different from those (creatinine: 0.45 ± 0.11 mg/dL; BUN: 47.19 ± 15.44 mg/dL, *n* = 8) in WT mice (creatinine: *P* = 0.8345; BUN: *P* = 0.3764, two-tailed *t*-test) (Fig. 2). Histological analysis revealed the absence of notable inflammatory infiltrates as well as the remarkable repair of tubular/nephron in CLU KO or WT kidneys after 14 days of IRI (Fig. 3). Under the microscopic views of both HE- and MT-stained sections, the kidneys from either KO or WT mice exhibited relatively intact tissues (tubular epithelium and interstitium) with the minimal levels of cellular infiltration (KO: 0.5–1; WT: 0.5) and interstitial fibrosis (KO: 0.5–1; WT: 0.5–2), which were mostly seen in the perivascular areas (Fig. 3). We also noticed that 2 out of 9 WT kidneys with ≥1.5 of fibrosis score showed some tubular dilatation and degeneration (reduced HE-stain of the cytoplasm) in outer area of renal cortex (Additional file 1: Figure S1).

### CLU deficiency decreases the function of the kidneys with atrophy

**Fig. 4:**
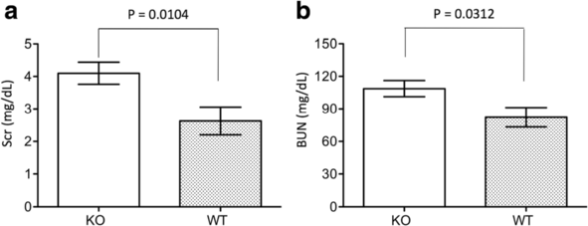
An association of CLU deficiency with worse kidney function after 30 days of IRI. On day 29 after IRI, contralateral kidneys were removed. After 24 h of surgery, the serum levels of both serum creatinine (Scr) and blood urea nitrogen (BUN) were measured. Data are presented as mean ± SEM of each group. **a** Scr in CLU KO group (*n* = 21) compared to WT control (*n* = 22), *p* = 0.00104 (two-tailed *t*-test). **b** BUN in CLU KO group (*n* = 16) compared to WT control (*n* = 11), *P* = 0.0312 (two-tailed *t*-test)

To investigate the role of CLU in the long-term effects of IRI on the kidney, the function of the kidneys with atrophy was examined in CLU KO mice (*n* = 21) compared with WT controls (*n* = 22) on day 30 after IRI. Because the serum volume of some mice was not sufficient for both measurements, the sample sizes for BUN were smaller (WT: *n* = 11; KO: *n* = 16). As shown in Fig. 4, the serum levels of both creatinine and BUN were higher in KO group than those in WT control, and were indicated by 4.1 ± 1.55 mg/dL of the creatinine in KO group (*n* = 21) compared to 2.63 ± 2 mg/dL in WT group (*n* = 22) (*P* = 0.0104, two-tailed *t*-test), and 108.71 ± 29.72 mg/dL of the BUN in KO group (*n* = 16) compared to 82.28 ± 29.34 mg/dL in WT group (*n* = 11) (*P* = 0.0312, two-tailed *t*-test). These data suggested that although the severity of renal atrophy was not affected by the deficiency in CLU expression (Fig. 1), the loss of kidney function was worse in CLU null kidneys with atrophy than that in WT controls after IRI.

### CLU deficiency increases cellular infiltration (CD8^+^ T cells and macrophages) in the kidneys with atrophy

**Fig. 5:**
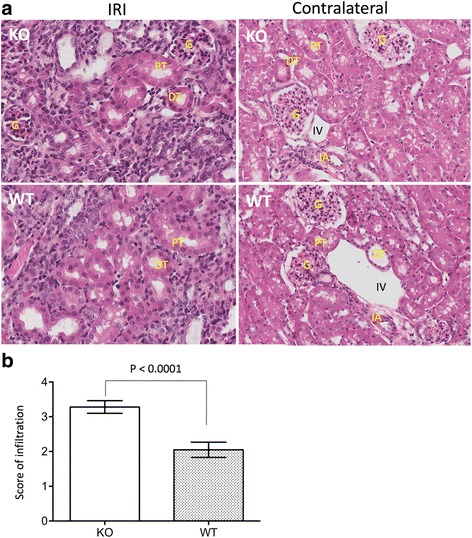
An association of CLU deficiency with worse cellular infiltration after 30 days of IRI. As described in Fig. 4, the contralateral right kidneys were harvested on day 29, and the injured left kidney on day 30 after IRI. The sections (two per each kidney) of both kidneys (the left and right from KO or WT) were stained with HE for the examination of the content of mononuclear infiltrates, labeled by *dark/black* nuclear staining. **a** A typical microscopic image of renal cortex in each group (KO: CLU null kidneys; WT: WT kidney sections). Left column: tubule damage and cellular infiltration of the injured left kidneys; right column: normal tissue architecture of the contralateral right kidneys. G: glomerulus; PT: proximal convoluted tubule; DT: distal convoluted tubule; IA: interlobular artery; and IV: interlobular vein. **b** The infiltration was semi-quantitatively scored in at least 20 randomly selected views in two separate sections of each injured left kidney, and was presented in average per view. Data are presented as mean ± standard derivation (SD) of each group. KO group vs. WT control: *P* < 0.0001 (two-tailed *t*-test, *n* = 8)

It has been documented previously that increased cellular infiltration is a long-term consequence of renal ischemia [41, 42], but was not significantly seen in the kidneys after 14 days of IRI in this study (Fig. 3). To understand the role of CLU expression in this pathologic change of the kidneys, the infiltrates of the kidneys with atrophy in the CLU KO group were compared to those in WT group after 30 days of IRI. The samples from each group were randomly selected for this study. As shown in Fig. 5, there was severe cellular infiltration in the left kidneys with atrophy in both groups, but not in contralateral kidneys. A semi-quantitative scoring showed that the severity of renal infiltration in KO group (3.28 ± 0.18) was higher than that in WT group (2.05 ± 0.22) (KO vs. WT, *P* < 0.0001, two-tailed *t*-test, *n* = 8) (Fig. 5b).

The phenotypes of infiltrates (CD4^+^, CD8^+^, B220^+^, pan NK^+^ CD11b^+^ and Mac3^+^) in the kidneys with atrophy were further examined using FACS analysis. Again, the samples for this study were randomly selected from each group, but were not the same as those for histological analysis in Fig. 5. As indicated in Table 1, similar to the higher infiltration scores in KO group by histological analysis (Fig. 5b), the total number of isolated infiltrates from CLU null kidneys was more than that in WT controls although the difference was not statistically significant in this limited number of samples (*P* = 0.0814, one-tailed *t*-test, *n* = 4). Statistically, only the percentages of both CD8^+^ and Mac3^+^ cells in KO group were significantly higher than those in WT group (CD8^+^ cells, *P* = 0.0377; Mac3^+^ cells, *P* = 0.0387, one-tailed *t*-test, *n* = 4), while the rest of phenotypes (CD4 T cells, B cell, NK cells, dendritic cells) were the same (Table 1). These data also indicated that the total numbers of infiltrating CD8 T cells and macrophages were more significantly higher in KO kidneys than those in WT controls. In addition, the percentage of each phenotype of leukocytes in the spleen from the same animal was also determined and used as a control for determining the specificity of kidney infiltrates. As shown in Table 2, there was no difference in the total numbers of splenocytes and the percentages of every phenotype between KO and WT groups. Taken together, these data suggested that CLU deficiency was associated with more cellular infiltrates, especially CD8^+^ (CD8 T cells) and Mac3^+^ cells (macrophages), in the kidneys with atrophy.

**Table 1:** The percentage of each phenotype of infiltrating immune cells in the kidneys

	WT (*n* = 4)	KO (*n* = 4)	*p* value (WT vs. KO)
Total isolated cells (× 10^6^)	20.39 ± 4.31	25.57 ± 4.78	0.0814
CD4 (%)	1.37 ± 0.91	2.38 ± 1.21	0.1547
CD8 (%)	3.51 ± 2.48	5.79 ± 3.76	0.0377
CD45R/B220 (%)	0.83 ± 0.62	1.08 ± 0.42	0.2853
Mac-3 (%)	4.22 ± 4.72	8.56 ± 5.21	0.0387
CD11b (%)	6.83 ± 1.43	8.02 ± 1.68	0.2195
pan-NK (%)	1.65 ± 0.68	2.4 ± 0.97	0.1384

Four kidneys/animals were randomly selected from each group for FACS analysis of infiltrating T cells (CD4^+^ and CD8^+^ cells), B cells (CD45R/B220^+^ cells), macrophages (Mac-3^+^ cells), dendritic cells (CD11b^+^ cells) and natural killer (NK) cells (pan-NK^+^ cells) on day 30 after IRI. The data were presented as mean ± SD, and were compared using one-tailed *t*-test

**Table 2:** The percentage of each phenotype of immune cells in the spleens

	WT (*n* = 4)	KO (*n* = 4)	*p* value (WT vs. KO)
Total isolated splenocytes (× 10^6^)	369.36 ± 177.5	373.5 ± 169.31	0.4871
CD4 (%)	15.85 ± 1.34	16.43 ± 1.92	0.3095
CD8 (%)	6.67 ± 1.56	8.13 ± 1.8	0.1839
CD45R/B220 (%)	59.63 ± 6.38	56.03 ± 6.37	0.2275
Mac-3 (%)	1.84 ± 0.42	1.37 ± 0.41	0.1620
CD11b (%)	5.67 ± 1.48	6.27 ± 1.38	0.2460
pan-NK (pan) (%)	3.41 ± 0.32	3.19 ± 0.33	0.1182

The spleens were collected from the same animals as described in Table 1, and the phenotypes of their splenocytes were determined using the FACS analysis, showing T cells (CD4^+^ and CD8^+^ cells), B cells (CD45R/B220^+^ cells), macrophages (Mac-3^+^ cells), dendritic cells (CD11b^+^ cells) and natural killer (NK) cells (pan-NK^+^ cells). The data were presented as mean ± SD, and were compared using one-tailed *t*-test

### CLU deficiency increases the damage of renal tubular integrity in the kidney with atrophy

**Fig. 6:**
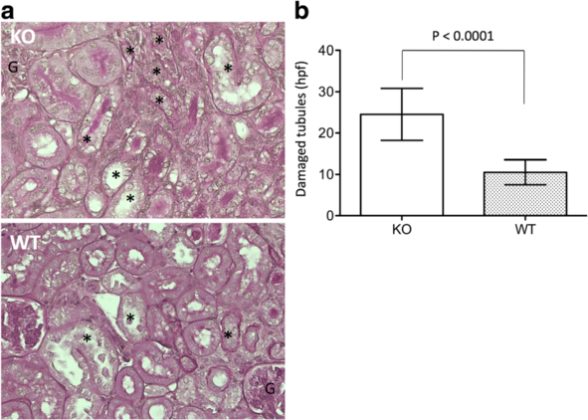
An association of CLU deficiency with more renal tubular injury after 30 days of IRI. The sections of CLU null and WT kidneys, harvested after 30 days of IRI, were stained with periodic acid-Schiff (PAS), and the number of injured tubules, including cellular loss (atrophy), intratubular cast formation, tubular cell flattening and vacuolation, in renal cortex was counted in each microscopic view (200x magnification). **a** Typical microscopic views of kidney sections in each group (KO: CLU KO kidneys; WT: WT kidneys). G: glomerulus; *: damaged tubules. **b** The number of injured tubules was counted in at least 20 randomly selected views in two separate sections of each kidney and was presented in average per view. Data are presented as mean ± SD of each group. KO group vs. WT control: *P* < 0.0001 (two-tailed *t*-test, *n* = 8)

Our previous studies have demonstrated that CLU deficiency worsens renal injury after IRI and impairs its repair during the early phase of IRI [26, 33]. However, its impact on the maintenance of the nephron of the kidney with atrophy after IRI was not known. After 30 days of IRI, histological analysis revealed that there were fewer intact tubules in the kidneys with atrophy in the CLU KO group compared with those in the WT group [24.52 ± 6.3 damaged tubules per hpf vs 10.52 ± 3.03 respectively (*P* < 0.0001, two-tailed *t*-test, *n* = 8)] (Fig. 6). These data suggested that CLU deficiency resulted in more severe renal tubular damage over the long term after IRI.

### CLU deficiency worsens tissue fibrosis in the kidney with atrophy

**Fig. 7:**
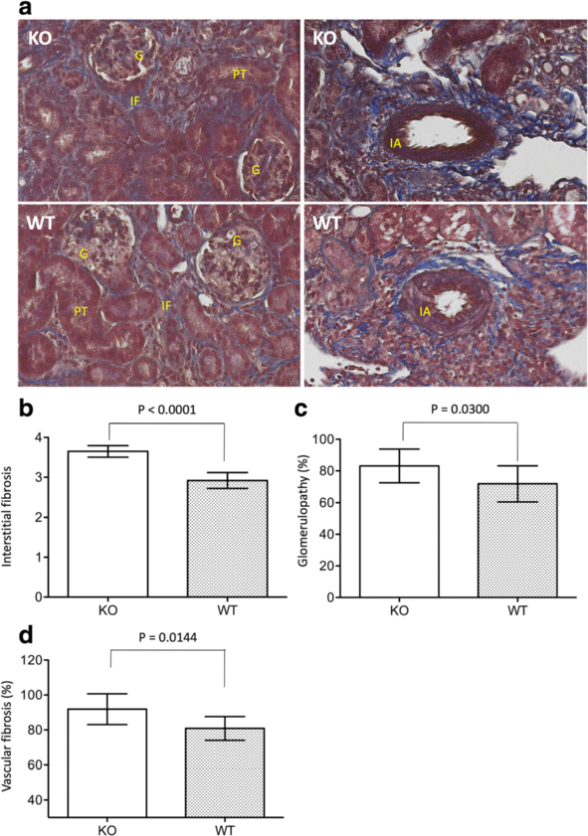
An association of CLU deficiency with more renal fibrosis (tubulointerstitial fibrosis, glomerulopathy and vascular fibrosis) after 30 days of IRI. The sections of CLU KO and WT kidneys, harvested after 30 days of IRI, were stained with MT. **a** Typical microscopic views of kidney sections in each group (KO: CLU KO kidneys; WT: WT kidneys), showing blue stain of collagen accumulation in the tubulointerstitial area, inside the glomerulus and interlobular arterial wall, and in the perivascular space. G: glomerulus; PT: proximal convoluted tubule; IA: interlobular artery; IF: interstitial fibrosis. **b** The extent of tubulointerstitial fibrosis was semi-quantitatively scored in at least 20 randomly selected views in two separate sections of each kidney and was presented in average per view. Data are presented as mean ± SD of each group. KO group vs. WT control: *P* < 0.0001 (two-tailed *t*-test, *n* = 8). **c** The percentage of affected glomeruli (glomerulopathy), including glomerulosclerosis (focal and seqmental sclerosis) and glomerular hypertrophy, was counted in two separate sections of each kidney, and the range of 180 to 250 glomeruli of each kidney was examined. Data are presented as mean ± SD of each group. KO group vs. WT control: *P* = 0.0300 (one-tailed *t*-test, *n* = 8). **d** The percentage of affected interlobular arterioles or arteries, determined by the presence of the fibrous intima, was counted in two separate sections of each kidney, and the range of 10 to 20 interlobular arteries or arterioles of each kidney was examined. Data are presented as mean ± SD of each group. KO group vs. WT control: *P* = 0.0144 (two-tailed *t*-test, *n* = 8)

In addition to cellular infiltration (Fig. 5), progression of fibrosis is recognized as one of long-term consequences of IRI in the kidney [43, 44]. To evaluate the effect of CLU deficiency on IRI-initiated renal fibrosis, the degree of tubulointerstitial fibrosis in the cortex, the percentage of affected glomeruli (glomerulopathy) and of interlobular arterioles or arteries of the kidneys in CLU KO mice were compared to those in WT controls. As shown in Fig. 7, abnormal collagen accumulation (blue stain) was seen in the tubulointerstitium, glomeruli, interlobular arterial wall and perivascular spaces of both CLU KO and WT kidneys. Semi-quantitative scoring showed that the renal fibrosis in KO group was significantly worse in all three areas (interstitial fibrosis, glomerulopathy, and vascular fibrosis) as compared to that in WT group. The interstitial fibrosis score of CLU null kidneys was 3.65 ± 0.15 and statistically higher than 2.92 ± 0.2 of WT controls (*P* < 0.0001, two-tailed *t*-test, *n* = 8) (Fig. 7b). Similar results were seen in the assessment of both glomerulopathy (*P* = 0.0300, one-tailed *t*-test, *n* = 8) and vascular fibrosis (*P* = 0.0144, two-tailed *t*-test, *n* = 8) in these two groups (Fig. 7c and d).

**Fig. 8:**
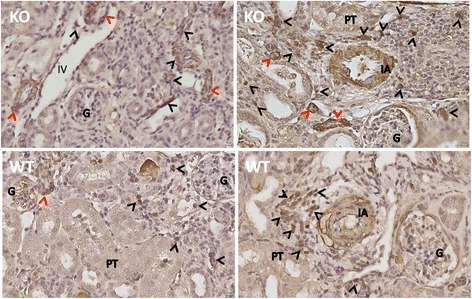
An association of CLU deficiency with more positivity in α-SMA stain. The expression of α-SMA (a myofibroblast marker) in the sections of CLU null and WT kidneys, harvested after 30 days of IRI, was examined by using a routine immunohistochemical method. Data were a typical microscopic view of the renal cortex in each group (KO: CLU KO kidneys; WT: WT kidneys), showing dark brown stain of α-SMA-expressing cells in the tubulointerstitial area and tubular epithelium, inside the glomerulus and interlobular arterial wall, and in the perivascular space. G: glomerulus; PT: proximal convoluted tubule; IA: interlobular artery; and IV: interlobular vein. *Black arrows*: infiltrating α-SMA^+^ cells; *red arrows*: tubular α-SMA^+^ cells

The α-SMA-expressing myofibroblasts plays an important role in the progression of renal fibrosis in different conditions including AKI [45, 46]. To further confirm the results from MT stain, the myofibroblast population, identified by α-SMA expression, was examined in CLU null kidneys compared to WT controls by using immunohistochemical stain. As shown in Fig. 8, two types of renal cells were found α-SMA positivity: infiltrates that were mainly localized in the interstitial and perivascular areas, and injured tubular epithelial cells. Furthermore, there were more α-SMA positive cells, especially tubular epithelial cells, in the sections of CLU null kidneys than in WT controls, which were consistent with the more collagen accumulation seen by MT-stained sections. Taken together, these data suggested that CLU deficiency worsened IRI-initiated fibrosis in the kidneys.

### CLU deficiency associates with down-regulation of EGF and up-regulation of CCL12 and MMP9

To further understand the molecular pathways by which CLU deficiency worsens IRI-induced fibrosis in the kidney, the expression of 84 fibrosis-related genes in CLU null kidneys compared to WT controls, also randomly selected from each group (*n* = 4), was examined using PCR array (Additional file 2: Table S1). Surprisingly, the expression of well-characterized pro-fibrogenic factors, such as transforming growth factor (TGF)-β1 and connective tissue growth factor (CTGF), was not correlated with an increase of fibrosis in CLU KO kidneys. As indicated in Table 3, by using a cutoff line (>2-fold change, *P* < 0.05), the expression levels of five genes in CLU null kidneys compared to WT controls (*n* = 4) were significantly changed after 30 days of IRI, which were the down-regulation of EGF as well as up-regulation of CCL12, Col3a1, MMP9 and TIMP1. Also, it was noticed that a significant increase (1.3–1.7 fold change, *p* < 0.05) was seen in the expression of AKT1, IL-1α, ITGB1, MMP14, PDGFA and TGIF1 (Additional file 2: Table S1). All these data indicated that these genes/pathways might mainly mediate the biological impacts of CLU deficiency on the initiation and progression of renal fibrosis and chronic inflammation after ischemic AKI.

**Table 3:** The change (>2-fold change, *p* < 0.05) of fibrosis-related genes in CLU KO kidneys compared to WT controls after 30 days of IRI

Gene (protein)	Fold change in KO kidneys vs. WT controls	*p* value (*n* = 4)	Main functions
*Egf* (Epidermal growth factor)	−2.7208	0.0341	The growth of epidermal and epithelial cells, and some of fibroblasts
*ccl12* (Chemokine (C-C Motif) ligand 12)	2.1439	0.0130	The recruitment of monocytes, lymphocytes and fibrocytes
*col3a1* (Collagen, Type III, alpha 1)	2.0162	0.0457	Fibril forming
*mmp9* (Matrix metallopeptidase 9)	2.573	0.0132	The degradation of the extracellular matrix
*timp1* (Tissue inhibitor of metalloproteinase 1)	2.0831	0.0260	Inhibition of the matrix metalloproteinases

Four renal tissues (cortex) were randomly selected from each group for PCR array analysis of gene expression using mouse fibrosis PCR array (Catalog No. PAMM-120Z). Negative in fold change: down-regulated; positive in fold change: up-regulated

## Discussion

Recent literature suggests that the full recovery of renal function after AKI is suboptimal because the long-term effects of AKI are associated with development of CKD, worsening of preexisting CKD and progression to ESRD [8, 47, 48]. Thus, understanding of the pathways mediating the long-term pathologic effects of AKI may eventually lead to develop a strategy for reducing or preventing the incidence of CKD or ESRD. Our previous studies have demonstrated that CLU deficiency worsens IRI and delays injury repair in adult kidneys [22, 26, 33]; however, the role of CLU deficiency in the long-term effects of ischemic AKI has not been investigated. The present study demonstrates that although CLU expression does not prevent renal atrophy after IRI, CLU deficiency is associated with the loss of renal function, more damage in the renal tubules and an increase in both cellular infiltrates (particularly CD8^+^ T cells and Mac3^+^ macrophages) and tissue fibrosis. All these negative impacts of CLU deficiency on the kidney after IRI may be mainly mediated by a cascade network of down-regulation of EGF and up-regulation of CCL12, Col3a1, MMP9 and TIMP1.

During the early or initial phase of AKI, sub-lethal injury of both tubular epithelial and endothelial/vascular cells is seen along with acute inflammation, followed by cellular repair and organ integrity re-establishment until functional recovery [2, 15, 49]. However, even after kidney function returns to baseline, patients are still at increased risk for the development of CKD after 2.5–3.4 years [47, 50]. The AKI-to-CKD transition is confirmed in rodent models of ischemic AKI and 3–4 weeks after IRI, the kidneys exhibit remarkable accumulation of tissue fibrosis [51–54]. Currently, the pathophysiology of AKI to CKD or AKI-initiated renal fibrosis is not completely clear, especially in humans, but experimental studies have identified several mechanisms, such as activation of memory or effector T cells and M2 macrophages [17, 54, 55], infiltration of bone marrow-derived myofibroblasts [51, 53], endothelial injury with vascular rarefaction and hypoxia [16, 17, 56, 57], and epigenetic changes and G2/M cell cycle arrest in epithelial cells [17, 58]. All of these components (pro-inflammatory immune cells, myofibroblasts, vascular rarefaction/hypoxia, and tubular epithelial cells) may play different roles during the transition from AKI to CKD in a cascade manner, but how they interact with each other remains for further investigation. Recently, Tanaka et al. [16] have proposed a central role of hypoxia in the AKI to CKD transition, and have hypothesized that after functional recovery from the early phase of AKI, the expression of vascular factors, such as vascular endothelial growth factor (VEGF) in tubular cells, is not yet restored, which results in capillary rarefaction leading to renal hypoxia, and consequently activation of inflammatory reactions, induces myofibroblast migration and/or differentiation and damages the regenerated tubules. All of these events together lead to tubulointerstitial fibrosis [16]. The primary role of hypoxia is supported by the fact that the density of blood vessels decreases by almost 45 % after 4 weeks of ischemic AKI [56], and treatment with VEGF preserves microvascular density and prevents renal fibrosis [59]. In this study, however, by PCR array analysis the expression of VEGF and EDN 1 (endothelin 1) was not different between CLU KO and WT kidneys (Additional file 2: Table S1), suggesting that the more severe fibrosis in CLU null kidneys may not be related to the hypoxia/vascular rarefaction, or the hypoxia is not a major factor for the CLU deficiency-related renal fibrosis after IRI.

It has been demonstrated that IRI initiates progressive renal weight or volume loss accompanying with tubular cell death (both apoptosis and necrosis) and interstitial infiltration [37–39], and our group and others using WT mice compared with CLU negative controls (CLU KO mice) have demonstrated that CLU is an anti-apoptotic or a prosurvival molecule in the kidney against IRI [22, 26, 33, 40]. In the present study of the transition of IRI to renal fibrosis, the data show that CLU deficiency does not affect the kidney weight or atrophy (Fig. 1), but it is associated with a decrease in the number of intact tubules (Fig. 6), and at the same time with an increase in the cellular infiltrates in the interstitial spaces (CD8 T cells and macrophages) and α-SMA^+^ myofibroblasts (Figs. 5, 7 and 8), suggesting that the survival and/or proliferation of different kidney cells, particularly tubular epithelial cells versus (myo) fibroblasts, may be regulated by CLU differently. Also, we showed that up-regulation of MMP9, TIMP1 and perhaps the membrane-type MMP14 was found in CLU KO kidneys after IRI. In general, MMP9 directly degrades extracellular matrix and is produced by a range of immune cells (e.g. lymphocytes and macrophages), and other cells including fibroblasts and endothelial cells following the exposure to pro-inflammatory cytokines (e.g. TNF-α, IFN-γ and IL-1β) [60], which is in agreement with the increase in cellular infiltrates, particularly CD8^+^ T cells and macrophages, and perhaps IL-1α within the CLU KO kidneys. More interestingly, MMP9 produced by renal tubular cells, macrophages and myofibroblasts contributes to renal fibrogenesis via osteopontin cleavage, which in turn recruits macrophage and induces renal tubular epithelial-mesenchymal transition (EMT) [61, 62], suggesting that MMP9 but not TGF-β is a major fibrotic factor for fibrosis in CLU KO kidneys. The major biological function of TIMPs including TIMP1 is to inhibit MMPs by forming a 1:1 enzyme-inhibitor complex, but the role of TIMP1 in kidney fibrosis has not been well investigated as of today. TIMP1 binds to proMMP9 to forms a specific complex [63], that may play a role in proMMP9 activation. Also TIMP1 exhibit other cellular activities, such as stimulation of cell proliferation of keratinocytes [64] and other cells [65]. However, the underlying mechanism by which TIMP1 involves in renal fibrosis remains further investigation.

Based on the evidence in literature, it is postulated that CLU can restrain the transition of IRI to renal fibrosis in the kidney by several mechanisms. First, complement activation plays a role in the progression of fibrosis in the kidney, as previous studies have shown that inhibition of complement activation reduces chronic inflammatory injury to renal tubular cells [66, 67] and endothelial cells [68], which is associated with reduced tissue fibrosis [66, 68]. CLU is a major glycoprotein in all the physiological fluids including the plasma [21], and this secreted form of CLU inhibits lytic terminal complement cytotoxicity in many early studies [29, 69, 70]. Thus, CLU in the blood and/or locally secreted by renal epithelial cells in WT mice may inactivate the complement during the transition of IRI to tissue fibrosis, which at least partially reduces leukocyte inflammation (both CD8 T cells and macrophagies) and tubular atrophy. Secondly, intracellular CLU is a cytoprotective protein that inhibits cell apoptosis by interacting with BAX or GRP78 [71–73], or promotes cell survival by activating Akt and NF-kB pathway [74, 75] and prosurvival autophagy [40, 76]. CLU expression has been confirmed in cultured glomerular mesangial and glomerular epithelial cells, and tubular epithelial cells [26, 40, 77–79], and facilitates cell survival following the exposure to pro-inflammatory cytokines (IFN-γ and TNF-α) [26] and hypoxia [40]. Thus, any of these CLU-expressing kidney cells in WT kidneys may have better survival than those in CLU KO counterparts under the chronic inflammatory attack and/or hypoxia, resulting in the prevention of nephron damage as seen in Fig. 6. Finally, the lack of CLU expression in tubular epithelial cells causes cell cycle arrest at G2/M phase in vitro [33], and worsens fibrosis in the kidneys after IRI in this study. These data may be consistent with a previous study, showing that c-jun NH_2_-terminal kinase (JNK) signaling mediates the cell cycle arrest at G2/M phase in proximal tubular cells, and treatment with a JNK inhibitor, or bypassing the G2/M arrest by administration of a p53 inhibitor rescues renal fibrosis after IRI [58]. Therefore, the potential of the down-regulation of EGF, G2/M phase interruption and more cell death probably result in the poorer maintenance of epithelial integrity of the nephron in the kidneys of CLU KO mice after IRI, which may contribute to more function loss (Fig. 4) as well as more space for infiltrates and myofibroblasts within the CLU null kidneys.

Myofibroblasts are commonly considered as the predominant effector cells in kidney fibrosis [80, 81], and a recent study using multiple genetically engineered mice has identified that a half of renal myofibroblasts are from local resident fibroblasts through proliferation, and the other half are derive through differentiation from bone marrow (35 %), the endothelial-to-mesenchymal transition (EndMT) (10 %) and EMT (5 %) [45], suggesting that renal fibrogenesis is involved in multiple cell types and multiple biological processes. The present study showed that more myofibroblasts were seen in CLU KO kidneys (Fig. 8), which is confirmed by PCR array, showing the higher transcript expression of Acta2 (α-SMA, 1.3753 fold increase with *P* = 0.0848), collagen (Col3a1), and fibrotic MMP9 [62] along with up-regulation of CCL12 (Table 3) and perhaps PDGF (a fibroblast growth factor). CCL12 stimulates the proliferation of infiltrating fibrocytes/bone marrow-derived myofibroblasts [82, 83], suggesting that a high level of CCL12 benefits the expansion of this myofibroblast population in CLU null kidneys. More interestingly, by immunohistochemical stain many tubules in these CLU KO kidneys are stained positively with α-SMA (Fig. 8), suggesting an important role of EMT in renal fibrosis in these kidneys. However, little is known about CLU expression and its function in renal fibroblast proliferation and survival, and myofibroblast differentiation from all different origins, which remain further investigation.

## Conclusions

The transition of AKI to CKD has major clinical significance in patients, but its molecular pathways are not fully understood. CLU is a chaperone-like protein and has different activities in different cells or experimental systems [22]. Our previous studies have demonstrated that CLU plays an important role in the maintenance and restoration of renal tubular integrity during the early phase of IRI [26, 33]. The data from current study indicate that as compared to CLU-expressing controls CLU deficiency has negative impacts on the preservation of kidney function and its normal architecture during the transition of IRI to renal fibrosis or CKD development. Also, our PCR array reveals that MMP9 but not TGF-β may be a major fibrotic factor for CLU deficiency-induced renal fibrosis. However, further understanding of the molecular mechanisms underlying the negative impacts of CLU deficiency on the kidney is needed, which may lead to develop an effective strategy to reduce the incidence of CKD after AKI.

## Additional files

Additional file 1: Figure S1. Tubular dilatation and degeneration in outer area of renal cortex of some WT kidneys. The sections of WT kidneys (≥ 1.5 of fibrosis score) were stained with MT (top) or H&E (middle and bottom). Data were presented as a typical microscopic image of renal cortex in each type of stain (MT or H&E stained sections), showing the tubular dilatation and degeneration in the squared area of the renal cortex. (TIFF 9328 kb) Additional file 2: Table S1. Fibrosis-related gene expression in CLU KO kidneys compared to WT controls after 30 days of IRI. (DOCX 21 kb)

## Abbreviations

AKI: Acute kidney injury
ANOVA: Analysis of variance
BUN: Blood urea nitrogen
CCL12: Chemokine (C-C motif) ligand 12
CKD: Chronic kidney disease
CLU: Clusterin
Col3a1: Collagen, Type III, alpha 1
CTGF: Connective tissue growth factor
EDN: Endothelin
EGF: Epidermal growth factor
EMT: Epithelial-mesenchymal transition
ESRD: End-stage renal disease
FACS: Fluorescence-activated cell sorting
HE: Hematoxylin and eosin
IFN: Interferon
IL: Interleukin
IRI: Ischemia-reperfusion injury
JNK: c-jun NH_2_-terminal kinase
KO: Knockout
MMP: Matrix metallopeptidase
MT: Masson’s trichrome
PAS: Periodic acid-Schiff
PBS: Phosphate buffered saline
PDGF: Platelet-derived growth factor
SD: Standard derivation
SMA: Smooth muscle actin
TBS: Tris buffer saline
TGF: Transforming growth factor
TIMP1: Tissue inhibitor of metalloproteinase 1
TNF: Tumor necrosis factor
UUO: Unilateral ureteral obstruction
VEGF: Vascular endothelial growth factor
WT: Wild type
