# Supplementary figures and images for: Relationship of clusterin with renal inflammation and fibrosis after the recovery phase of ischemia-reperfusion injury
Source: BMC Nephrol. 2016 Sep 20;17:133. doi: 10.1186/s12882-016-0348-x (PMC5028988; doi:10.1186/s12882-016-0348-x)

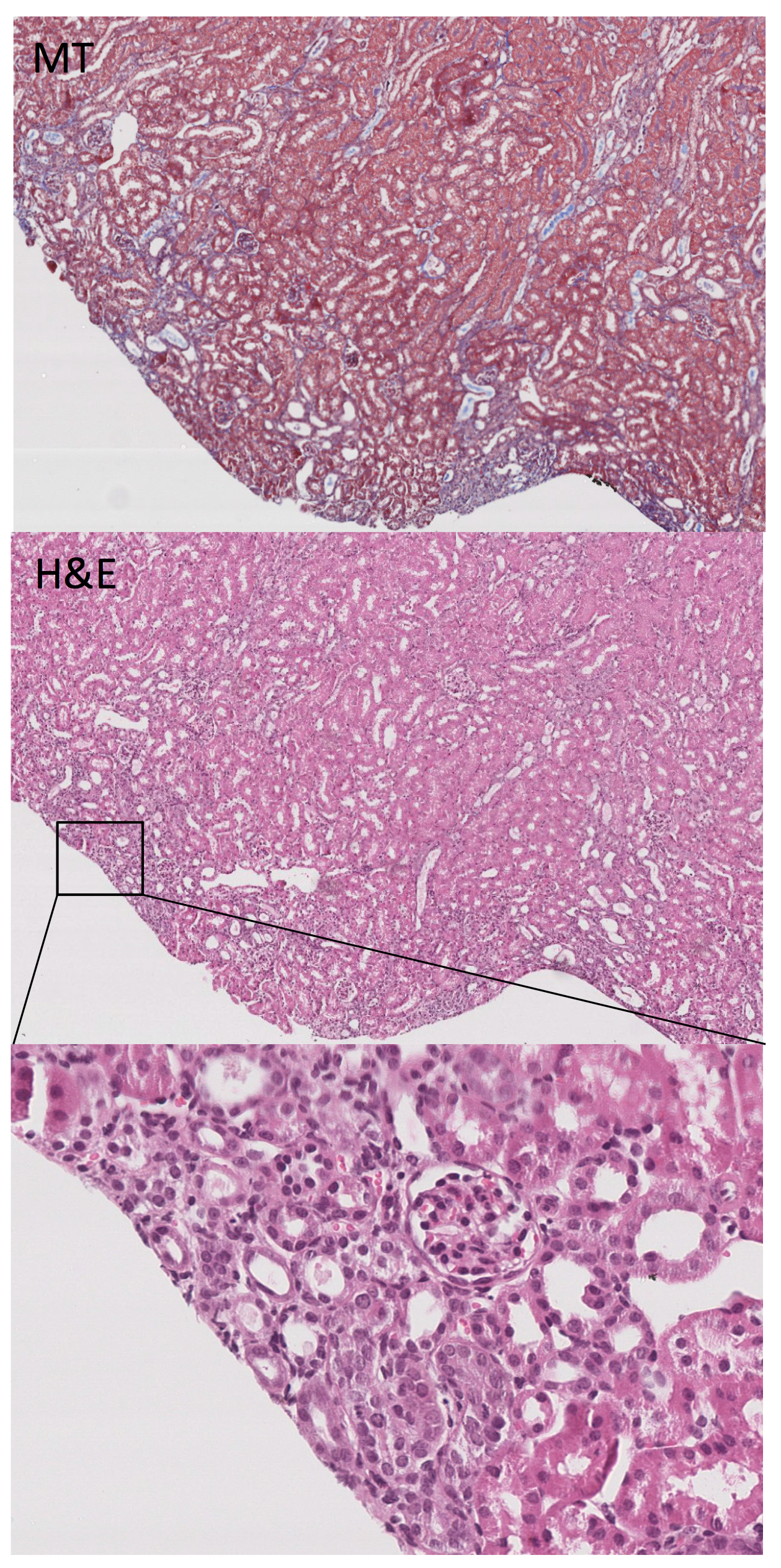

Supplement: Additional file 1: Figure S1. — Tubular dilatation and degeneration in outer area of renal cortex of some WT kidneys. The sections of WT kidneys (≥ 1.5 of fibrosis score) were stained with MT (top) or H&E (middle and bottom). Data were presented as a typical microscopic image of renal cortex in each type of stain (MT or H&E stained sections), showing the tubular dilatation and degeneration in the squared area of the renal cortex. (TIFF 9328 kb) [file 12882_2016_348_MOESM1_ESM.tiff]
