# Supplementary material for: Relationship of clusterin with renal inflammation and fibrosis after the recovery phase of ischemia-reperfusion injury
Source: BMC Nephrol. 2016 Sep 20;17:133. doi: 10.1186/s12882-016-0348-x (PMC5028988; doi:10.1186/s12882-016-0348-x)
Supplement: Additional file 2: Table S1. — Fibrosis-related gene expression in CLU KO kidneys compared to WT controls after 30 days of IRI. (DOCX 21 kb) [file 12882_2016_348_MOESM2_ESM.docx]

Table 1. Fibrosis-related gene expression in CLU KO kidneys compared to WT controls after 30 days of IRI

| Gene | Fold Change | P value | Functional Gene Grouping | | | | | | | |
| --- | --- | --- | --- | --- | --- | --- | --- | --- | --- | --- |
|  |  |  | PF | AF | EM&CA | IC&C | GF | ST | EMT | A |
| *Acta2* | 1.3753 | 0.084833 | √ |  |  |  |  |  |  |  |
| *Agt* | -1.0033 | 0.918926 | √ |  |  |  | √ |  |  |  |
| *Akt1* | 1.3434 | 0.021694 |  |  |  |  |  |  | √ |  |
| *Bcl2* | -1.1282 | 0.328044 |  |  |  |  |  |  |  | √ |
| *Bmp7* | -1.0695 | 0.597594 |  | √ |  |  |  | √ | √ |  |
| *Cav1* | 1.1088 | 0.349025 |  |  |  |  |  | √ |  |  |
| *Ccl11* | 1.0505 | 0.582561 | √ |  |  | √ |  |  |  |  |
| *Ccl12* | 2.1439 | 0.013004 | √ |  |  | √ |  |  |  |  |
| *Ccl3* | 1.3913 | 0.627928 | √ |  |  | √ |  |  |  |  |
| *Ccr2* | 1.4333 | 0.257799 |  |  |  | √ |  |  |  |  |
| *Cebpb* | 1.2301 | 0.901033 |  |  |  |  |  | √ |  |  |
| *Col1a2* | 1.7406 | 0.149148 |  |  | √ |  |  |  | √ |  |
| *Col3a1* | 2.0162 | 0.04573 |  |  | √ |  |  |  | √ |  |
| *Ctgf* | -1.1094 | 0.247186 | √ |  |  |  | √ |  |  |  |
| *Cxcr4* | -1.0808 | 0.591017 |  |  |  | √ |  |  |  |  |
| *Dcn* | 1.3577 | 0.17714 |  |  |  |  |  | √ |  |  |
| *Edn1* | 1.2974 | 0.432485 |  |  |  |  | √ |  |  |  |
| *Egf* | -2.7208 | 0.034110 |  |  |  |  | √ |  |  |  |
| *Eng* | -1.0956 | 0.473619 |  |  |  |  |  | √ |  |  |
| *Fasl* | 1.6226 | 0.168885 |  |  |  |  |  |  |  | √ |
| *Grem1* | -1.6447 | 0.266852 | √ |  |  |  |  | √ |  |  |
| *Hgf* | -1.1226 | 0.767279 |  | √ |  |  | √ |  |  |  |
| *Ifng* | -1.1169 | 0.438985 |  | √ |  | √ |  |  |  |  |
| *Il10* | 1.21 | 0.963996 |  | √ |  | √ |  |  |  |  |
| *Il13* | 1.5399 | 0.128268 | √ |  |  | √ |  |  |  |  |
| *Il13ra2* | 1.3165 | 0.605412 | √ | √ |  | √ |  |  |  |  |
| *Il1a* | 1.6277 | 0.033128 |  |  |  | √ |  |  |  |  |
| *Il1b* | 1.6511 | 0.367377 |  |  |  | √ |  |  |  |  |
| *Il4* | 1.1911 | 0.672479 |  |  |  |  |  |  |  |  |
| *Il5* | 1.1194 | 0.350763 |  |  |  |  |  |  |  |  |
| *Ilk* | 1.2478 | 0.125759 |  |  |  |  |  |  |  |  |
| *Inhbe* | -1.3275 | 0.272036 |  |  |  |  |  | √ |  |  |
| *Itga1* | 1.038 | 0.711509 |  |  | √ |  |  |  |  |  |
| *Itga2* | 1.3766 | 0.355373 |  |  | √ |  |  |  |  |  |
| *Itga3* | -1.0762 | 0.707938 |  |  | √ |  |  |  |  |  |
| *Itgav* | 1.0432 | 0.766048 |  |  | √ |  |  |  | √ |  |
| *Itgb1* | 1.3652 | 0.031178 |  |  | √ |  |  |  | √ |  |
| *Itgb3* | -1.0691 | 0.930175 |  |  | √ |  |  |  |  |  |
| *Itgb5* | 1.2382 | 0.354944 |  |  | √ |  |  |  |  |  |
| *Itgb6* | 1.0923 | 0.666844 |  |  | √ |  |  |  |  |  |
| *Itgb8* | 1.0063 | 0.716804 |  |  | √ |  |  |  |  |  |
| *Jun* | 1.1941 | 0.679478 |  |  |  |  |  | √ |  |  |
| *Lox* | 1.8091 | 0.23993 |  |  | √ |  |  |  |  |  |
| *Ltbp1* | -1.215 | 0.269325 |  |  |  |  |  | √ |  |  |
| *Mmp13* | 1.0455 | 0.967724 |  |  | √ |  |  |  |  |  |
| *Mmp14* | 1.7046 | 0.046896 |  |  | √ |  |  |  |  |  |
| *Mmp1a* | -1.7222 | 0.217027 |  |  | √ |  |  |  |  |  |
| *Mmp2* | 1.3572 | 0.466027 |  |  | √ |  |  |  | √ |  |
| *Mmp3* | 1.4953 | 0.192237 |  |  | √ |  |  |  | √ |  |
| *Mmp8* | 1.3696 | 0.423731 |  |  | √ |  |  |  |  |  |
| *Mmp9* | 2.573 | 0.01319 |  |  | √ |  |  |  | √ |  |
| *Myc* | 1.2107 | 0.416308 |  |  |  |  |  | √ |  |  |
| *Nfkb1* | 1.362 | 0.095804 |  |  |  |  |  | √ |  |  |
| *Pdgfa* | 1.6094 | 0.026102 |  |  |  |  | √ |  |  |  |
| *Pdgfb* | 1.2852 | 0.603938 |  |  |  |  | √ |  |  |  |
| *Plat* | 1.4392 | 0.098583 |  |  | √ |  |  |  |  |  |
| *Plau* | -2.0189 | 0.285398 |  |  | √ |  |  |  |  |  |
| *Plg* | -3.4958 | 0.225359 |  |  | √ |  |  |  |  |  |
| *Serpina1a* | -1.6211 | 0.110806 |  |  | √ |  |  |  |  |  |
| *Serpine1* | 1.4745 | 0.47374 |  |  | √ |  |  |  | √ |  |
| *Serpinh1* | 1.0982 | 0.779599 |  |  | √ |  |  |  |  |  |
| *Smad2* | 1.1602 | 0.302837 |  |  |  |  |  | √ | √ |  |
| *Smad3* | -1.3539 | 0.746856 |  |  |  |  |  | √ |  |  |
| *Smad4* | -1.0722 | 0.918468 |  |  |  |  |  | √ |  |  |
| *Smad6* | 1.271 | 0.324785 |  |  |  |  |  | √ |  |  |
| *Smad7* | -1.0939 | 0.797427 |  |  |  |  |  | √ |  |  |
| *Snai1* | -1.0865 | 0.594685 | √ |  |  |  |  |  | √ |  |
| *Sp1* | 1.02 | 0.777 |  |  |  |  |  | √ |  |  |
| *Stat1* | -1.1615 | 0.508913 |  |  |  |  |  | √ |  |  |
| *Stat6* | 1.4877 | 0.058961 |  |  |  |  |  | √ |  |  |
| *Tgfb1* | 1.2676 | 0.622968 |  |  |  |  |  | √ | √ |  |
| *Tgfb2* | -1.0277 | 0.978369 |  |  |  |  |  | √ | √ |  |
| *Tgfb3* | -1.6869 | 0.194958 |  |  |  |  |  | √ | √ |  |
| *Tgfbr1* | -1.5186 | 0.453912 |  |  |  |  |  | √ |  |  |
| *Tgfbr2* | -1.2689 | 0.344811 |  |  |  |  |  | √ |  |  |
| *Tgif1* | 1.6172 | 0.027416 |  |  |  |  |  | √ |  |  |
| *Thbs1* | -1.7682 | 0.264253 |  |  |  |  |  | √ |  |  |
| *Thbs2* | 1.242 | 0.535464 |  |  |  |  |  | √ |  |  |
| *Timp1* | 2.0831 | 0.025953 |  |  | √ |  |  |  | √ |  |
| *Timp2* | 1.6054 | 0.104125 |  |  | √ |  |  |  |  |  |
| *Timp3* | -1.679 | 0.14486 |  |  | √ |  |  |  |  |  |
| *Timp4* | -1.0377 | 0.877311 |  |  | √ |  |  |  |  |  |
| *Tnf* | 1.1866 | 0.543321 |  |  |  | √ |  |  |  |  |
| *Vegfa* | -1.0135 | 0.865174 |  |  |  |  | √ |  |  |  |

The levels of fibrosis-related genes were analyzed using mouse fibrosis PCR array (Catalog No. PAMM-120Z, QIAGEN, Toronto, ON, Canada). Positive in fold change: up-regulated; negative in fold change: down-regulated; PF: pro-fibrotic; AF: anti-fibrotic; EM&CA: extracellular matrix and cell adhesion; IC&C: inflammatory cytokines &chemokines; GF: growth factors; ST: signal transduction; EMT: epithelial-to-mesenchymal transition; A: apoptosis; “✓”: member of a functional group. Red highlight: > 2-fold change or p < 0.05.
